# Book Reviews

**Published:** 1908-03

**Authors:** 


### BOOK REVIEWS.

---

SYPHILIS.—A treatise for practitioners, By Edward L. Keyes, Jr., A. B., M. D., Ph. D. Clinical Professor of Genito-Urinary Surgery, New York Polyclinic Medical School and Hospital; Lecturer on Surgery, Cornell University Medical School; Surgeon to St. Vincent's Hospital. With sixty-nine illustrations in the text and nine plates, seven of which are colored. D. Appleton & Co., New York.

The wide experience of Dr. Keyes and his father makes this book on syphilis one of exceptional value. The views regarding syphilis, although not altogether orthodox, are sane and sensible. The book, as stated in the preface, is founded largely upon the observation of 2,500 cases of syphilis seen during the last forty years in the office of Dr. Keyes, and for twenty years previously in that of Dr. Van Buren, to whose offices the Keyes succeeded. With this retrospect of sixty years, we naturally expect a practical work. This Dr. Keyes has given us, and to it has added much that is of interest, and of recent observation of the spirochaeta pallida, the spiral shaped micro-organism discovered by Schaudinn, and now believed to be the cause of lues. A number of good cuts are reproduced, and the various staining methods described. In regard to the etiological role of the spirochaeta pallida, the author says: "So overwhelming, indeed, is the mass of evidence that in spite of the fact that the organism has not been cultivated, it seems certain that the cause of syphilis has at least been found, and we may safely say that the spirochaeta of Schaudin is either the cause of syphilis or one phase in the life cycle of some micro-organism which is the cause of syphilis."

In the treatment insoluble injections are very highly spoken of, and Keyes says that where mercury will work at all, these injections are always as efficient as other methods, and often much more so. He has seen lesions yield to them after having resisted every other form of treatment, even soluble injections. It may be safely predicted that it will be many years before a more reliable book on syphilis will be published.

GONORRHEA—ITS DIAGNOSIS AND TREATMENT, By Frederick Baumann, Ph. D., M. D., Professor of Genito-Urinary Diseases in the Reliance Medical College, and Instructor in Dermatology in the College of Physicians and Surgeons, Chicago. Fifty-two illustrations in the text. D. Appleton & Co., New York.

As a monograph on the use of urethral dilators, this work may be considered a very successful and valuable book. As a short monograph on gonorrhea or as a book of practical value to the student, we think it a failure.

In the first place, the author's view point is too limited. He clings too closely to Kollmann and Oberlaender, and omits too much of proven value along other lines.

He dogmatically claims that no method of treatment will prevent the occurrence of relapses, and from the complacency with which he views them, we feel inclined to think his plan of treatment is probably productive of many of these relapses which might be prevented if a less active method were used—in fact it is nothing unusual, by other methods now in vogue to carry these conditions to successful termination without a relapse.

The descriptions of the pathological conditions are good, and usually ahead of the treatment.

We cannot agree that internal medication, given with irrigation treatment, does not seem to have a beneficial result, as is claimed by this author; nor has it been our experience that the urethra has been kept irritated by the balsams. On page 110 it is recommended that liquids be cut down so as to concentrate the urine and enhance the value of the balsams. To this point we cannot subscribe, as we believe overloaded concentrated urine will produce more urethral irritation than the effect of the balsams, thus produced, justify.

---

Climbing from crib into his mother's bed, one morning a small boy squeezed his mother's mammary gland and said, "Honk! Honk! Mama, you are my automobile."

